# Spatially Heterogeneous Responses of Body Growth to El Niño‐Southern Oscillation Signal During Different Life Stages in Jumbo Squid (*Dosidicus gigas*)

**DOI:** 10.1002/ece3.74148

**Published:** 2026-08-06

**Authors:** Mingfeng Jiang, Fan Zhang, Sisong Dong, Jun Yu, Ming Shao, Xinjun Chen

**Affiliations:** ^1^ College of Marine Living Resource Sciences and Management Shanghai Ocean University Shanghai China; ^2^ Key Laboratory of Oceanic Fisheries Exploration, Ministry of Agriculture and Rural Affairs Shanghai Ocean University Shanghai China; ^3^ National Engineering Research Center for Oceanic Fisheries Shanghai Ocean University Shanghai China; ^4^ Key Laboratory of Sustainable Exploitation of Oceanic Fisheries Resources, Ministry of Education Shanghai Ocean University Shanghai China

## Abstract

Climate variability can affect growth differently across life stages, but whether these effects vary among spatially distinct groups remains poorly understood. We investigated how climate variability experienced at different life stages interacts with spatial context to shape the growth trajectories of jumbo squid (*Dosidicus gigas*) in the eastern Pacific Ocean. We analyzed a dataset comprising statolith‐based age estimates for 1415 female jumbo squid collected over multiple years. Spatial growth patterns were modeled using nonlinear mixed‐effects growth models, and life stage‐specific effects of the El Niño–Southern Oscillation (ENSO) were assessed using generalized additive models based on Niño 1 + 2 sea surface temperature anomalies experienced from birth to 8 months of age. The Schnute growth model with spatial random effects on growth rate provided the best fit. Equatorial squid grew faster, matured earlier, and exhibited lower interindividual variability than southern squid. Early in life, ENSO effects on growth were broadly consistent across individuals, with cold phases associated with enhanced growth and warm phases associated with reduced growth. During mid‐to‐late ontogeny, however, this pattern reversed, with cold phases negatively and warm phases positively affecting growth. This reversal occurred earlier in equatorial individuals than in southern individuals. These findings demonstrate that climate exposure at different developmental stages has ontogenetically variable effects on growth in *D*. *gigas*, shifting from a cold‐phase growth advantage early in life to a warm‐phase advantage later in development. The timing of this shift differs between equatorial and southern groups, reflecting spatial contrasts in thermal regimes and life‐history schedules. These asynchronous reversals in ENSO responses demonstrate that climate–growth relationships cannot be generalized across either space or development and highlight the need for spatially explicit management of migratory marine species.

## Graphical abstract

Climate exposure at different developmental stages exerts ontogenetically variable effects on growth in *Dosidicus gigas* , with ENSO impacts shifting from cold‐phase growth advantages in early life to warm‐phase advantages later in development. The timing of this shift differs between equatorial and southern groups, reflecting spatial contrasts in thermal regimes and life‐history schedules. These asynchronous reversals in ENSO responses demonstrate that climate–growth relationships cannot be generalized across either space or development, highlighting the need for spatially explicit management of migratory marine species.

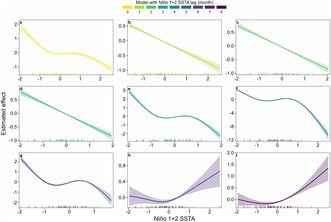

## 1 Introduction

Climate change is reshaping marine ecosystems worldwide, with measurable and intensifying impacts on marine organisms over the past century, affecting individual physiology, survival, and population dynamics (Denechaud et al. 2020). Growing evidence indicates that climatic conditions experienced during different life stages can exert persistent, stage‐specific effects on growth by shaping developmental trajectories that extend across the entire life span (Dahlke et al. 2020; Jonsson and Jonsson 2014). Over recent decades, the intensity of anomalous climate events has increased (Perkins‐Kirkpatrick and Lewis 2020), with the frequency projected to rise further in the coming decades (Chung et al. 2024; Wang et al. 2024), potentially amplifying climatic stress experienced by marine organisms. Under such conditions, understanding how marine species respond to climate exposure across life stages and elucidating the mechanisms underlying these responses has become increasingly important for interpreting growth variability in natural populations.

Widely distributed marine species experience a wide range of environmental conditions across their geographic distributions and therefore often exhibit pronounced spatial variation in growth patterns (Gertseva et al. 2017). For example, in the South Pacific, *Thunnus alalunga* display faster growth in eastern regions compared with western regions (Williams et al. 2012). Similarly, Jackson et al. (2003) reported marked spatial heterogeneity in size at maturity of the *Nototodarus gouldi* in southern Australian waters. Such spatial variation in growth has commonly been attributed to regional differences in environmental conditions, particularly sea temperature and food availability (Brandt et al. 1992; Mason and Brandt 1996).

Life stage–specific environmental effects on performance in marine species remain poorly understood, particularly during early life stages (Jonsson and Jonsson 2014). Early life represents a critical window during which environmental conditions can exert disproportionate and persistent influences on individual growth and development through physiological and energetic constraints (Fonseca and Cabral 2007). Importantly, identical environmental conditions can produce markedly different, and even opposing, effects depending on whether individuals experience them early or later in ontogeny (Forster and Hirst 2012). For widely distributed and highly migratory species, cohorts originating from different spawning habitats and migrating along distinct routes may be exposed to similar large‐scale climate events yet exhibit divergent growth responses later in life, reflecting spatial heterogeneity in earlier environmental conditions and the developmental trajectories they shape.

The jumbo squid ( *Dosidicus gigas* ) is a highly migratory oceanic species of substantial economic importance, distributed widely across the eastern Pacific from the coastal waters of Alaska to those off Chile (Arkhipkin, Argüelles, et al. 2015). Jumbo squid exhibits pronounced spatial structuring in phenotypic traits, with smaller‐sized individuals predominating in tropical regions and medium to large‐sized individuals more common in high‐latitude waters (Frawley et al. 2019). This spatial variation in body size suggests substantial regional differences in growth trajectories rather than differences in age structure alone. Notably, spawning grounds of jumbo squid are also spatially segregated, occurring from tropical waters to high‐latitude temperate waters (Gilly et al. 2006; Jiang et al. 2024), which implies that cohorts originating from different regions are exposed to distinct environmental conditions during life stages, potentially predisposing individuals to divergent growth responses later in life.

Among the dominant sources of climate variability in the eastern Pacific, the El Niño–Southern Oscillation (ENSO) plays a central role in shaping the environmental conditions and has been shown to exert strong effects on the growth of the jumbo squid (Fang et al. 2024; Watters et al. 2003). ENSO can affect jumbo squid growth through both temperature‐ and food‐mediated pathways, with effects that may originate during early ontogeny and propagate across subsequent life‐history stages. Arkhipkin, Rodhouse, et al. (2015) demonstrated that sea temperatures experienced at multiple life stages influence longevity in jumbo squid off Peru, highlighting the importance of thermal conditions throughout development. Portner et al. (2020) showed that ENSO‐driven warming in the Gulf of California alters diet composition and prey availability, suggesting that food limitation during El Niño events contributes to reduced growth. Despite these insights, the extent to which spatial variation in growth trajectories of jumbo squid is shaped by ENSO exposure across life stages remains poorly understood. To address this knowledge gap, we examined spatial growth patterns and life‐history stage–specific ENSO effects on individual late growth of jumbo squid using an extensive age‐structured dataset spanning multiple years. Our objectives are: (a) to identify the spatial differences in growth patterns of jumbo squid, and (b) to assess how ENSO exposures through life stages alter spatial growth patterns.

## 2 Materials and Methods

### 2.1 Sample Collection

**FIGURE 1:**
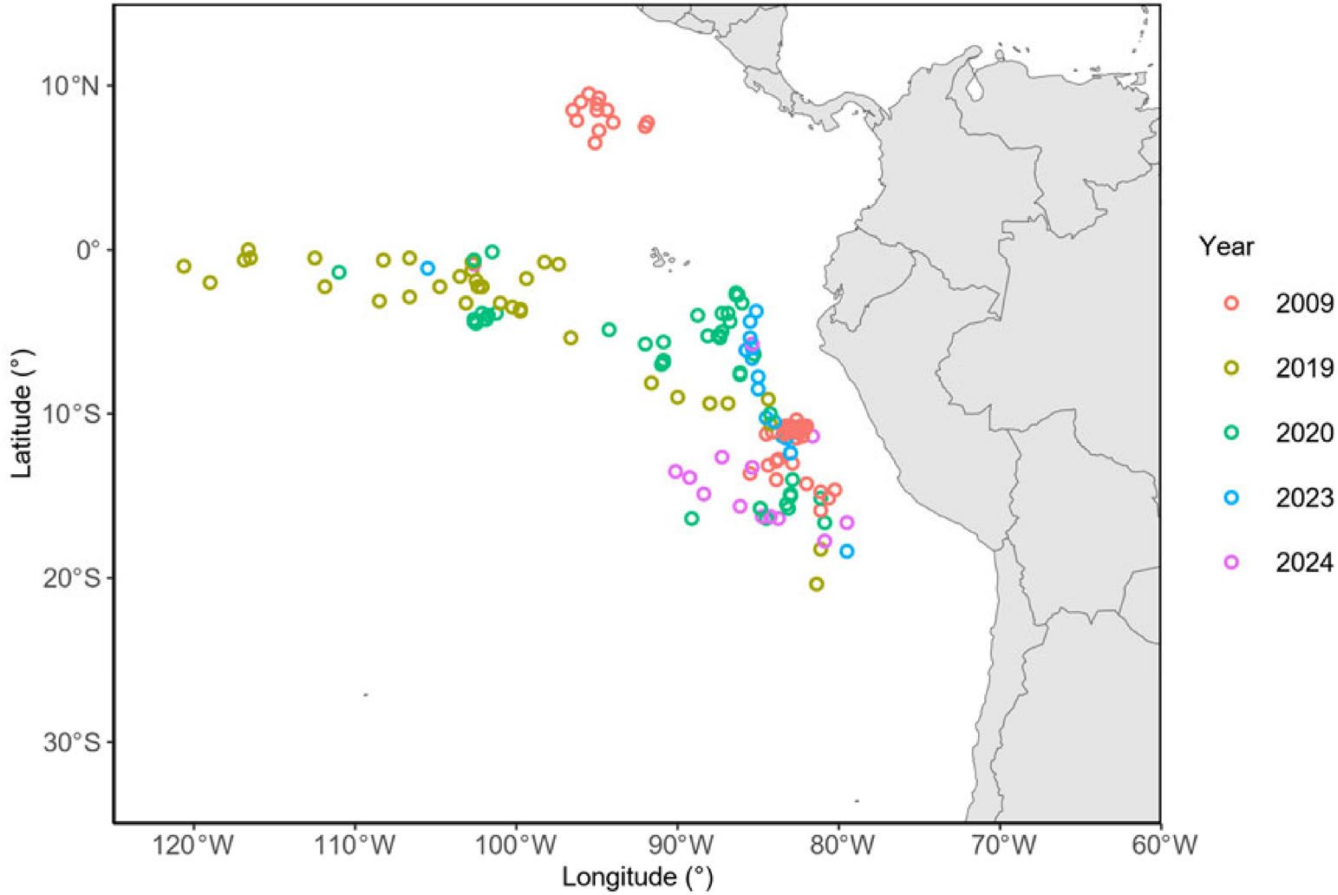
Spatial distribution of jumbo squid sampling locations in the Pacific across study periods.

Biological samples of the jumbo squid were collected from commercial jigging operations conducted in international waters of the eastern Pacific. A total of 1415 female individuals were sampled during multiple periods, including 2009, 2019–2020, and 2023–2024 (Figures S1 and S2). All specimens were frozen onboard immediately after capture and subsequently transported to the laboratory for further biological analyses. For each individual, mantle length (ML), body weight (BW), sex, and gonadal maturity stage were recorded. ML was measured to the nearest 1 mm, and BW was measured to the nearest 1 g. Gonadal maturity was classified into five stages (I–V) following established criteria (Nigmatullin and Markaida 2009). Based on sampling locations, all individuals were assigned to one of two spatial groups: the equatorial groups (10° N–10° S, EG) and the southern group (10° S–30° S, SG) (Figure 1).

**FIGURE 2:**
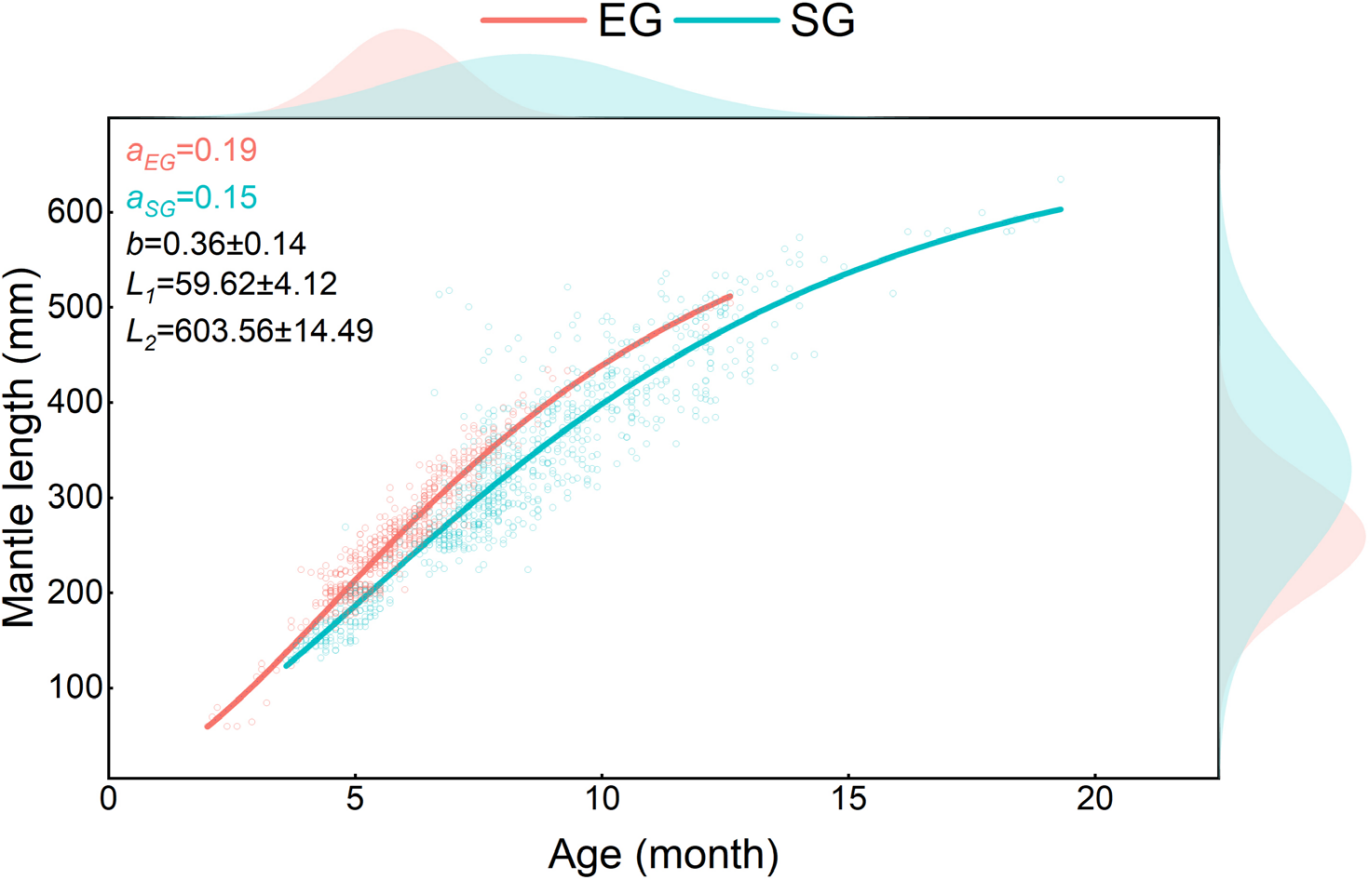
Age–mantle length relationships of jumbo squid estimated by the Schnute model with region‐specific random effects. EG, equatorial group; SG, southern group.

### 2.2 Statolith Analysis

Statoliths were removed from the statocysts, sequentially labeled, and immersed in 75% ethanol in 1.5‐mL centrifuge tubes to remove residual organic material and soft tissue adhering to the statolith surface. Subsequent embedding, grinding, and polishing followed the protocol described by Liu et al. (2011). Age determination was based on the assumption that statolith growth increments are deposited daily in jumbo squid, consistent with previous validation studies (Chen et al. 2011; Liu et al. 2017). Each statolith was independently counted by two readers. If the difference between the two counts was < 10% of their mean, the age estimate was accepted. Otherwise, the statolith was re‐examined by both readers, and the final age was calculated as the mean of four independent counts (Table S1).

### 2.3 Determination of Spatial Growth Patterns

Three growth models widely applied in cephalopod studies (the von Bertalanffy, Gompertz, and Schnute models) were used to fit mantle length data measured as described in Section 2.1 with age estimates derived in Section 2.2 (Goicochea‐Vigo et al. 2019; Patterson 1988; Zepeda‐Benitez et al. 2014). The basic formulations of the three growth models are presented below (Schnute 1981; von Bertalanffy 1957; Winsor 1932).

(1) $$L\left(t\right)=L_{\infty }^{\mathrm{vB}}\left(1-e^{-k^{\mathrm{vB}}\left(t-t_{0}^{\mathrm{vB}}\right)}\right)$$

(2) $$\begin{array}{c} L\left(t\right)=L_{\infty }^{\mathrm{Gom}}\exp\left[-\exp\left(-k^{\mathrm{Gom}}\left(t-t_{0}^{\mathrm{Gom}}\right)\right)\right] \end{array}$$

(3) $$\begin{array}{c} L\left(t\right)={\left[L_{1}^{b}+\left(L_{2}^{b}-L_{1}^{b}\right)\frac{1-e^{-a\left(t-t_{1}\right)}}{1-e^{-a\left(t_{2}-t_{1}\right)}}\right]}^{\frac{1}{b}} \end{array}$$

where t refers to age, and $L\left(t\right)$ refers to mantle length at age t. For the von Bertalanffy model, $L_{\infty }^{\mathrm{vB}}$ is the asymptotic length, $k^{\mathrm{vB}}$ is the growth coefficient, and $t_{0}^{\mathrm{vB}}$ represents the theoretical age at which length is zero. For the Gompertz model, $L_{\infty }^{\mathrm{Gom}}$ is the asymptotic length, $k^{\mathrm{Gom}}$ is the growth coefficient, and $t_{0}^{\mathrm{Gom}}$ denotes the time at which the growth rate is maximal. For the Schnute model, $L_{1}$ and $L_{2}$ denote reference lengths at ages $t_{1}$ and $t_{2}$, respectively, a is the growth rate parameter, and b determines the shape of the growth curve.

Growth patterns were fitted using nonlinear mixed‐effects models implemented in the nlme framework (Pinheiro et al. 2025). The sampling region was treated as a spatial grouping factor and incorporated as a random effect on growth parameters to evaluate alternative spatial growth structures. For the von Bertalanffy model, the parameter $t_{0}^{\mathrm{vB}}$ was fixed at a value approaching zero (−1 × 10^−16^), as it is often weakly informed by the data and difficult to estimate reliably, particularly when early life stages are under‐represented (Schemmel et al. 2022). In addition, we fitted alternative von Bertalanffy models in which $t_{0}^{\mathrm{vB}}$ was retained as an estimable parameter, to avoid reducing the credibility of our conclusions due to inconsistencies in model assumptions. For the Schnute model, the reference ages $t_{1}$ and $t_{2}$ were set to the minimum and maximum observed ages in the dataset (Schnute 1981). Specifically, a candidate set of models was constructed by allowing regional random effects to act on different combinations of parameters for each growth function, thereby quantifying spatial heterogeneity in growth patterns among regions. To accommodate heteroscedasticity in mantle length observations, residual variance was modeled using a power variance function dependent on fitted values, allowing variance to increase with body size (Pinheiro et al. 2025). In total, 3 baseline models without random effects and 25 models including spatial random effects were fitted and compared. Model selection was performed using the Akaike information criterion (AIC) and the Bayesian information criterion (BIC), with lower values indicating better model support (Burnham and Anderson 2004).

Instantaneous growth rate at age t was calculated as the first derivative of the best‐fitted growth curve with respect to age:

(4) $$\begin{array}{c} g\left(t\right)=\frac{dL\left(t\right)}{dt} \end{array}$$

Since analytical derivatives of nonlinear growth models can be numerically unstable, growth rates were approximated using finite differences with one‐sided differences applied near the boundaries of the observed age range:

(5) $$\begin{array}{c} g\left(t\right)\approx \frac{L\left(t+h\right)-L\left(t-h\right)}{2h} \end{array}$$

where h denotes a small age increment used to approximate the derivative of the growth curve via finite differences.

### 2.4 Effects of Climate Exposure Among Life Stages on Individual Growth

Normalized residuals of each individual $r_{i}$ were extracted from the best fitted growth model in Section 2.3 to account for heteroscedasticity induced by the fitted‐value–dependent variance structure. Specifically, residuals were scaled by the observation‐specific conditional standard deviation:

(6) $$\begin{array}{c} r_{i}=\frac{L_{i}-{\hat{L}}_{i}}{\hat{\sigma }{\left|{\hat{L}}_{i}\right|}^{\hat{\delta }}} \end{array}$$

where $L_{i}$ is the observed mantle length, ${\hat{L}}_{i}$ is the corresponding fitted value from the growth model, $\hat{\sigma }$ is the estimated residual standard deviation, and $\hat{\delta }$ is the power parameter of the fitted‐value–dependent variance function.

With Niño 1 + 2 sea surface temperature anomaly (SSTA) used as an indicator of ENSO variability (Jiang et al. 2024; Yu et al. 2017, Figure S3), we applied generalized additive models (GAMs) to examine the relationship between ENSO exposure at different life stages and subsequent individual growth. Normalized growth residuals derived were used as the response variable. For each individual, birth month and Niño 1 + 2 SSTA from the birth month to 8 months after birth, together with spatial region, were included as explanatory variables. A total of 9 GAMs were fitted separately, each corresponding to a specific lagged exposure window, as follows:

(7) $$\begin{array}{c} r_{i}=\beta_{0}+\alpha_{\mathrm{re}\left(i\right)}+\eta_{\mathrm{bm}\left(i\right)}+\theta_{\mathrm{re}\left(i\right),\mathrm{bm}\left(i\right)}+f_{\mathrm{re}\left(i\right)}\left(\mathrm{var}\right)+\varepsilon_{i} \end{array}$$

where $\beta_{0}$ is the overall intercept, $\mathrm{re}\left(i\right)$ indicates the spatial region to which individual *i* belongs, $\mathrm{bm}\left(i\right)$ is the birth month of individual *i*, var denotes explanatory variables including ${\text{SSTA}}_{i,k}$ (the Niño 1 + 2 SSTA corresponding to month k after the inferred hatching month of individual i), $s_{g}\left(\right)$ represents the region‐specific smooth function, and $\varepsilon_{i}$ is the individual‐level error term assumed to follow a Gaussian distribution. Accordingly, the sample size varied among GAMs, as individuals younger than the focal lag month were not included in the corresponding model.

All statistical analyses were performed in R4.4.0 (R Core Team 2024), with nonlinear growth models fitted using the nlme package (Pinheiro et al. 2025) and GAMs implemented using the mgcv package (Wood 2025).

## 3 Results

### 3.1 Spatial Growth Patterns of Jumbo Squid

Among the 25 candidate models incorporating spatial random effects, the Schnute growth model provided the best fit to the spatial growth patterns of jumbo squid, followed by the Gompertz model, whereas the von Bertalanffy model performed the poorest. The von Bertalanffy models with the parameter $t_{0}^{\mathrm{vB}}$ retained also performed worse than the Schnute models and the Gompertz models (Table S2). Similarly, all three baseline models without random effects exhibited poorer fits than their corresponding mixed‐effects formulations, with the same ranking in model performance (Schnute > Gompertz > von Bertalanffy) (Table 1). The best‐supported model was the Schnute model with a spatial random effect applied to the growth‐rate parameter 𝑎 (M16, AIC = 13,761, BIC = 13,798).

**TABLE 1:** Comparison of alternative nonlinear growth models with different spatial random effect structures for jumbo squid.

Model name	Curve type	Random effect	AIC	BIC
M01	von Bertalanffy	NA	14,198	14,219
M02	von Bertalanffy	$L_{\infty }^{\mathrm{vB}}$	14,061	14,082
M03	von Bertalanffy	$k^{\mathrm{vB}}$	14,063	14,084
M04	von Bertalanffy	$L_{\infty }^{\mathrm{vB}}$+$k^{\mathrm{vB}}$	14,058	14,089
M05	Gompertz	NA	14,059	14,117
M06	Gompertz	$t_{0}^{\mathrm{Gom}}$	13,779	13,810
M07	Gompertz	$k^{\mathrm{Gom}}$	13,969	14,001
M08	Gompertz	$L_{\infty }^{\mathrm{Gom}}$	13,771	13,803
M09	Gompertz	$t_{0}^{\mathrm{Gom}}+L_{\infty }^{\mathrm{Gom}}$	13,769	13,811
M10	Gompertz	$k^{\mathrm{Gom}}$+$L_{\infty }^{\mathrm{Gom}}$	13,774	13,816
M11	Gompertz	$k^{\mathrm{Gom}}$+$t_{0}^{\mathrm{Gom}}$	13,769	13,811
M12	Gompertz	$k^{\mathrm{Gom}}$+$t_{0}^{\mathrm{Gom}}$+$L_{\infty }^{\mathrm{Gom}}$	13,775	13,833
M13	Schnute	NA	14,017	14,054
M14	Schnute	$L_{1}$	13,771	13,808
M15	Schnute	$L_{2}$	13,777	13,814
M16	Schnute	a	13,761	13,798
M17	Schnute	b	13,766	13,803
M18	Schnute	$L_{1}$+a	13,765	13,813
M19	Schnute	$L_{1}$+b	13,761	13,807
M20	Schnute	$L_{2}$+a	13,765	13,813
M21	Schnute	$L_{2}$+b	13,761	13,807
M22	Schnute	a+b	13,761	13,808
M23	Schnute	$L_{1}$+$L_{2}$	13,763	13,811
M24	Schnute	$L_{1}$+$L_{2}$+a	13,771	13,834
M25	Schnute	$L_{1}$+$L_{2}$+b	13,765	13,823
M26	Schnute	$L_{1}$+a+b	13,766	13,829
M27	Schnute	$L_{2}$+a+b	13,766	13,829
M28	Schnute	$L_{1}$+$L_{2}$+a+b	13,767	13,820

*Note:* Overall, the EG exhibited faster growth (𝑎 = 0.19) than the SG (Table 1) (𝑎 = 0.15) (Figure 2). However, standardized growth residuals exhibited greater variability in the SG (mean ± SD = −0.004 ± 1.135) than in the EG (mean ± SD = 0.006 ± 0.756), indicating increased inter‐individual variation in growth for SG (Figure S4).

**FIGURE 3:**
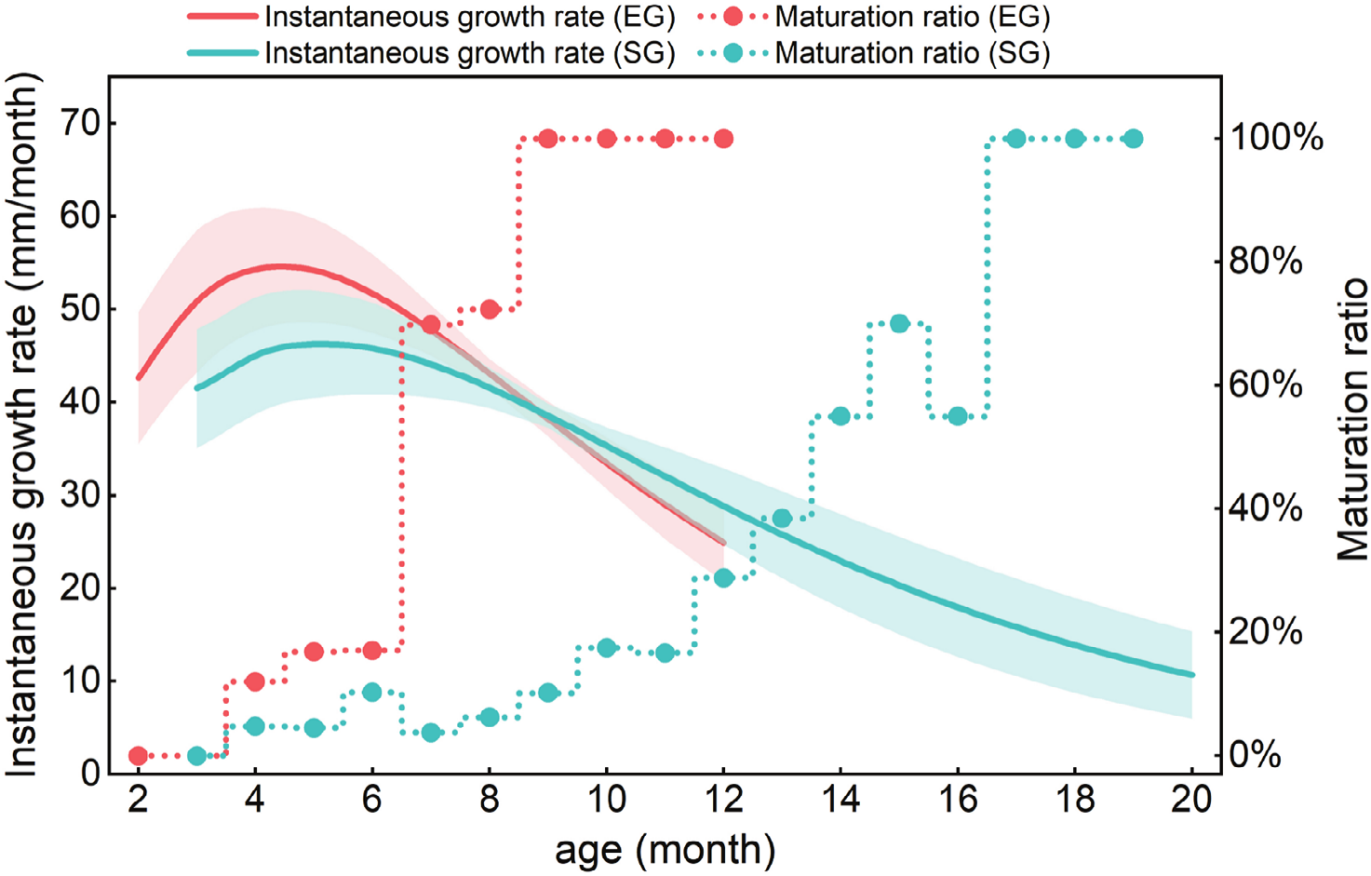
Age‐specific instantaneous growth rates and maturation ratio of the equatorial group and southern group. EG, equatorial group; SG, southern group.

Both EG and SG exhibited rapid early growth followed by a progressive slowdown at later ages (Figure 3). For EG, the instantaneous growth rate peaked at approximately 3 months of age, reaching values of 47.7–61.4 mm month^−1^, whereas the SG showed a later growth peak at around 4 months of age, with lower maximum growth rates ranging from 40.1 to 52 mm month^−1^.

Analysis of gonadal development revealed pronounced spatial differences in the timing of maturation (Figure 3). The EG reached full maturity substantially earlier, with 100% of individuals classified as mature from approximately 9 months of age onward, whereas complete maturation in the SG was not observed until around 17 months of age. Notably, while the progression of gonadal maturation in the EG followed an approximately logistic pattern, the SG exhibited a non‐monotonic pattern. The first peak occurred at around 6 months of age, where 10.3% of individuals were mature, followed by a second peak at approximately 15 months of age, where the proportion of mature individuals reached 70%.

### 3.2 Life Stage‐Specific Effects of Climate Exposure on Individual Development

**FIGURE 4:**
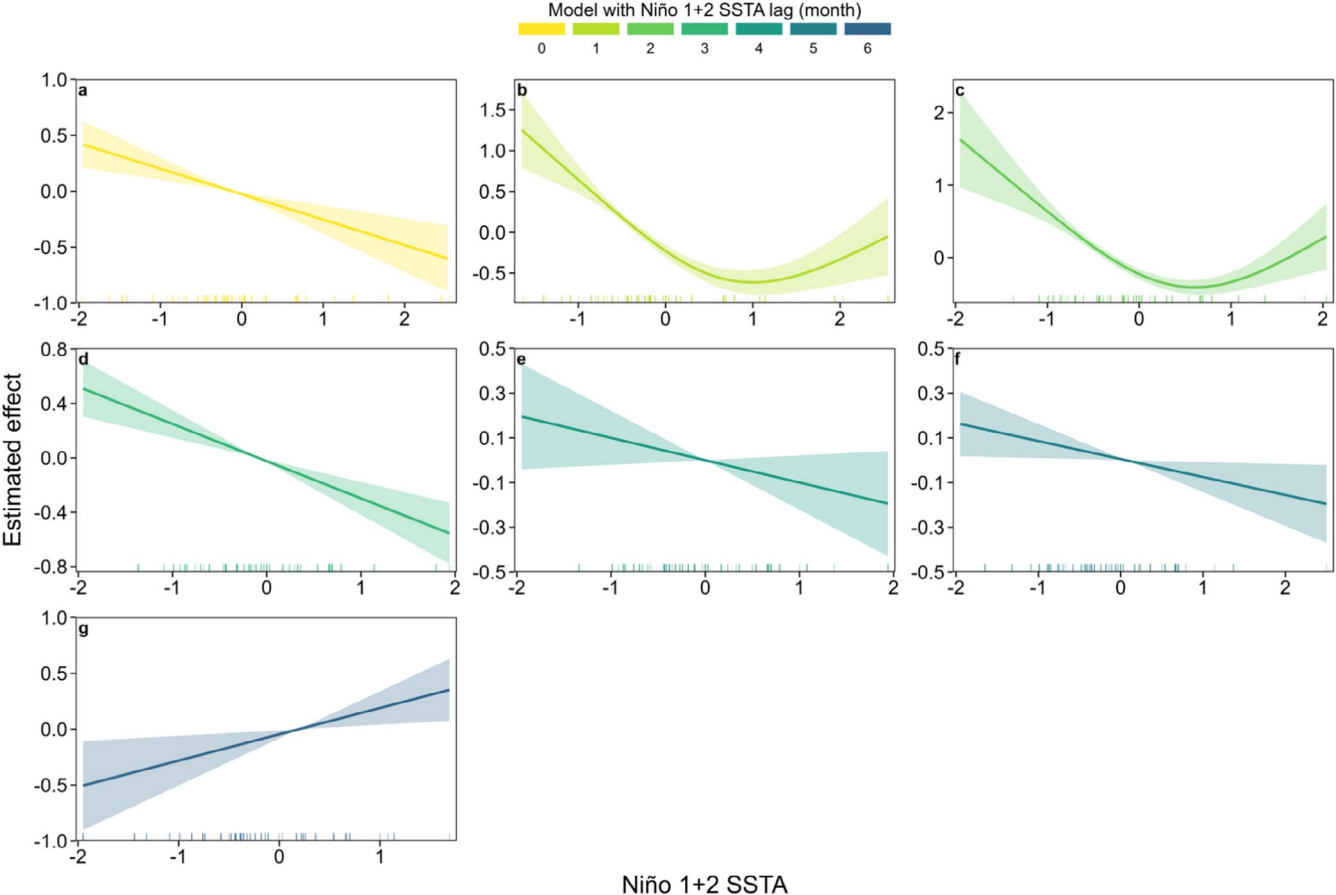
Spatially heterogeneous responses of normalized growth residuals to life stage‐specific Niño 1 + 2 SSTA in the equatorial waters. Notes: Panels (a–g) represent Niño 1+2 SSTA experienced at 0, 1, 2, 3, 4, 5, and 6 months after hatching.

The individual growth of jumbo squid responded differently to climatic exposure across life‐history stages, and these responses exhibited spatial heterogeneity. For the EG, significant associations were detected for ENSO conditions corresponding to months 0–3 after hatching, whereas statistical support weakened during months 4–6 and was absent during months 7–8 (Table S3). Exposure to the cold phase of ENSO during the early to mid life‐history stages (0–5 months) promoted individual growth (Figure 4a–f). During months 3–5, the growth response to ENSO gradually weakened, as indicated by a reduction in the slope of the response curves (Figure 4d–f). At month 6, the growth response reversed, with the warm phase becoming more favorable for individual growth (Figure 4g).

**FIGURE 5:**
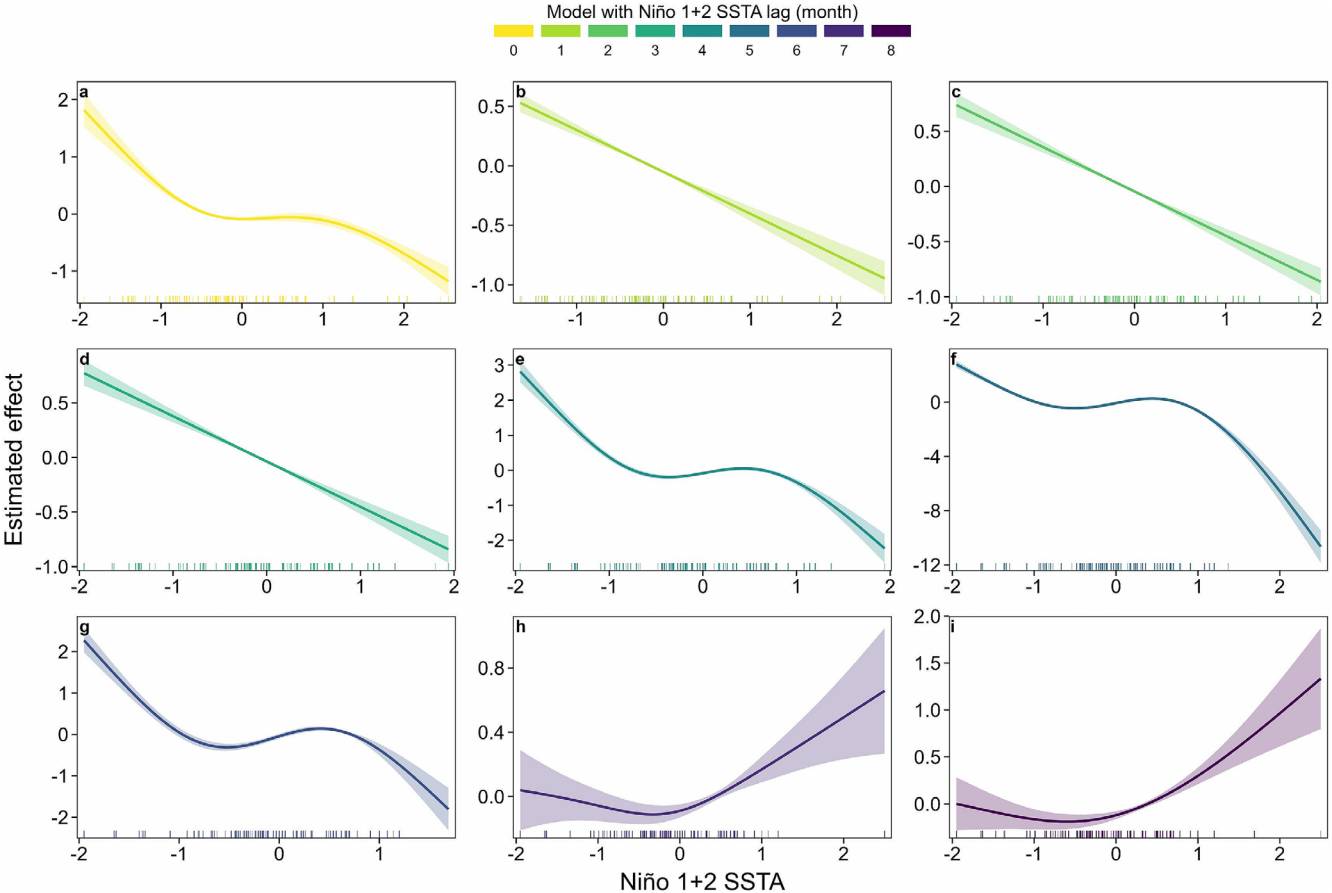
Spatially heterogeneous responses of normalized growth residuals to life stage‐specific Niño 1 + 2 SSTA in the southern waters. Notes: Panels (a–i) represent Niño 1+2 SSTA experienced at 0, 1, 2, 3, 4, 5, 6, 7, and 8 months after hatching.

For the SG, individual growth during the first 0–7 months was significantly associated with ENSO conditions experienced in each month of life (Table S3). Similar to the EG, exposure to the cold phase of ENSO during the early to mid‐stages favored individual growth; however, this growth‐promoting period lasted longer in the SG, extending from hatching through the first 7 months of life (Figure 5a–g). During the mid‐life‐history stage, the SG also exhibited a reversal in growth response, but this shift began at 7 months after hatching (Figure 5h–i), indicating a delayed reversal relative to the EG.

### 3.3 Temporal Effect on Individual Development

**FIGURE 6:**
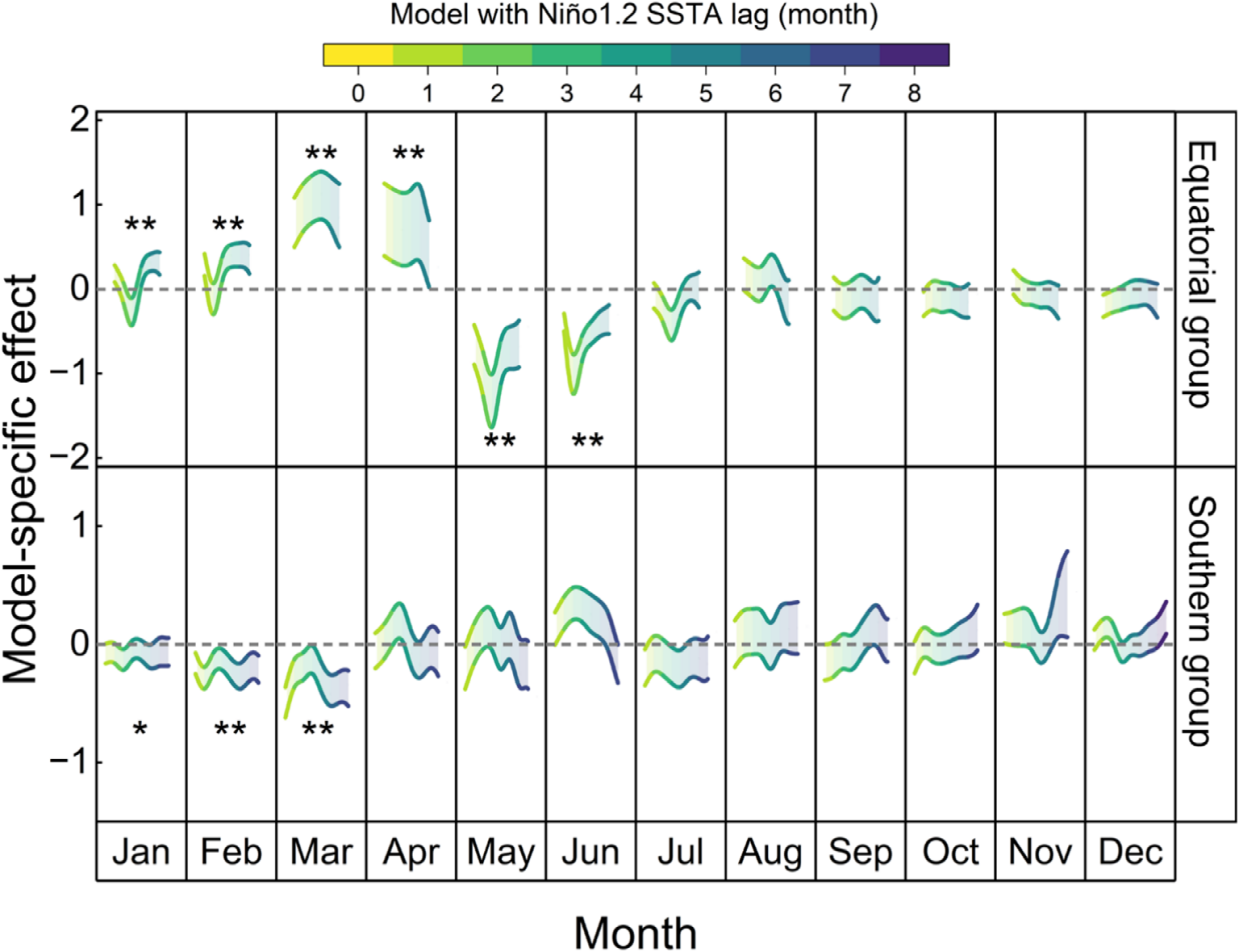
Spatially heterogeneous responses of normalized growth residuals to birth month in jumbo flying squid. Colored curves represent estimated birth‐month effects from separate models fitted with Niño1 + 2 SSTA lagged by 0–8 months. For each month, *p*‐values from all lag‐specific models were combined using Fisher's method. “*” denotes months with significant combined *p*‐values (*p* < 0.05), and “**” denotes months with significant combined *p*‐values (*p* < 0.01).

Across lag‐specific models, birth‐month effects showed a broadly consistent pattern, but the strength of evidence varied by months and by regions. For the EG, individuals hatched between January and April grew significantly better than expected (*p* < 0.01), whereas those hatched in May–June grew significantly worse than expected (*p* < 0.01) (Figure 6). In contrast, for the SG, individuals hatched in January–March grew significantly worse than expected (*p* < 0.05), while no significant birth‐month effect independent of the climate signal was detected for the remaining months (Figure 6).

## 4 Discussion

### 4.1 Spatial Heterogeneity in Growth Patterns of Jumbo Squid

We observed pronounced spatial heterogeneity in the growth trajectories of jumbo squid, with individuals from equatorial waters exhibiting faster growth and earlier maturation compared to those from higher latitudes. Such spatial differences likely arise from the contrasting hydrographic conditions between equatorial and southern waters, which impose distinct constraints on growth and life‐history strategies.

Latitudinal variation in growth and body size is well‐documented for widely distributed species, particularly marine ectotherms, reflecting temperature‐mediated differences in metabolic rates, growth duration, and energy allocation (Conover 1992). Jackson and Moltschaniwskyj (2001) reported latitudinal variation in growth of *Loliolus noctiluca* , with larger body size and longer lifespan at higher latitudes and the opposite pattern at lower latitudes. Similar latitudinal size patterns have also been observed in fish species, including *Gadus morhua* and *Mallotus villosus* (Hedeholm et al. 2010; Thorsen et al. 2010). Most squids do not cease somatic growth during gonadal development, with individual growth continuing until reproduction and subsequent death (Ceriola and Jackson 2010; McGrath and Jackson 2002). For jumbo squid inhabiting warm equatorial waters, elevated temperatures are likely to accelerate gonadal development (Han and Chen 2020), resulting in earlier maturation, shorter growth duration, and reproduction at smaller body sizes. In contrast, in cooler southern waters, slower gonadal development and delayed maturation extend the growth period and lifespan, thereby increasing the potential for individuals to attain larger body sizes. Together, these contrasting growth and maturation strategies provide a mechanistic explanation for the spatial heterogeneity in growth patterns observed in jumbo squid.

### 4.2 Life Stage–Specific ENSO Effects on Individual Growth

Growth deviations expressed at later stages therefore integrate the combined effects of environmental conditions experienced throughout ontogeny, with positive or negative growth responses at successive stages combining to shape individual growth trajectories. ENSO exposure during different life‐history stages interacts with spatial context to shape divergent growth responses of jumbo squid. During the early developmental stages, cold ENSO phases were generally associated with enhanced growth potential, whereas during subadult and adult stages, warmer ENSO phases were more favorable for individual growth. These contrasting responses reflect fundamental differences in ecological and physiological requirements across ontogeny, as organisms grow and progressively alter their environmental demands (Jonsson and Jonsson 2014).

Individuals from both EG and SG exhibited reversals in growth responses to ENSO during specific months, although the timing of these reversals differed between regions. Such patterns likely reflect ontogenetic shifts in thermal tolerance and changes in energy allocation associated with the onset of gonadal development. Pecl and Jackson (2008) suggested that the effects of temperature on squid life history may differ, and even oppose each other, between early and later developmental stages. Staaf et al. (2011) reported that the optimal sea temperature range for early life stages of jumbo squid is relatively low (approximately 15°C–25°C), whereas adults can tolerate sea temperatures of nearly 30°C. As individuals grow and gonads develop, thermal tolerance and metabolic capacity increase, and growth becomes less constrained by narrow thermal optima, allowing individuals to withstand higher temperatures (Dan et al. 2025). Elevated temperatures have also been shown to positively influence gonadal development in jumbo squid (Dan et al. 2022), which helps explain why individuals inhabiting warmer equatorial waters initiate gonadal development earlier than those from cooler waters. Although somatic growth in jumbo squid does not cease with the onset of gonadal development, the initiation of reproductive investment alters energy allocation patterns, with a greater proportion of assimilated energy directed toward gonadal tissues and a reduced proportion toward somatic tissues (Han et al. 2019; Lian et al. 2022). Elevated temperatures during gonadal development may facilitate growth in jumbo squid by increasing metabolic and feeding rates, thereby supporting greater energy allocation to somatic growth during this stage of continued growth and reproductive investment (Moltschaniwskyj and Martínez 1998).

Nevertheless, Niño 1 + 2 SSTA represents a large‐scale ENSO signal rather than the local thermal conditions directly experienced by each individual. Since jumbo squid are highly migratory and individual movement trajectories were unavailable, it was not possible to assign a specific local environmental history to each life stage (Jiang et al. 2026). In addition, the local expression of ENSO in the southern waters may be modified by variability in the Peru–Chile upwelling system, including changes in upwelling intensity, current structure, and marine productivity (Cooley et al. 2022). These regional processes may introduce uncertainty into the estimated magnitude and timing of the response.

### 4.3 Temporal Effects on Individual Growth

Our results indicate that, in the equatorial region, individuals hatched during January–April exhibit greater growth potential, whereas those hatched during May–June show lower growth potential. Individuals in the equatorial region are mainly hatched in waters off Central America, Ecuador, the coast of Panama, and offshore Costa Rica. During boreal winter (January–April), strong cross‐isthmus winds generate wind jets along the Central American coast, which enhance chlorophyll‐*a* concentrations and create favorable feeding grounds (Pennington et al. 2006). As these jets disappear in May, the conditions supporting high‐quality feeding grounds also decline, and individual growth potential decreases accordingly.

In the southern region, lower growth potential was detected only among individuals hatched during January–March, which may reflect the combined effects of higher summer temperatures and reduced productivity. Individuals in the southern region are generally considered to originate from spawning grounds along the Peruvian and Chilean coasts, where hydrographic conditions are dominated by the Peru Current system (Liu et al. 2011; Yu et al. 2025). Cooley et al. (2022) reported that the Peru–Chile upwelling system typically experiences anomalously high sea surface temperatures during summer. A common feature of these warm events is a marked weakening, or even reversal, of Ekman suction, which in turn reduces surface productivity (Espinoza‐Morriberón et al. 2025).

### 4.4 Influence on Stock Management

Ignoring spatial population structure can lead to misinterpretation of stock status and, ultimately, to ineffective fisheries management (Cadrin 2020). In the southeastern Pacific, jumbo squid has traditionally been managed as a single stock, largely due to weak genetic differentiation among regions and the administrative convenience of managing a highly migratory, transboundary species (Yu et al. 2025). Although single‐stock approaches are common in fisheries management, they can be problematic for species exhibiting pronounced spatial heterogeneity in growth patterns. In such cases, accurate assessment of population dynamics often requires spatially structured stock assessments or management strategy evaluation (MSE) frameworks that explicitly account for spatial variability (Correa et al. 2021). Spatially structured assessment approaches typically partition the stock's distribution into discrete spatial units, allowing each unit to follow its own growth and recruitment dynamics, which has been shown to substantially improve parameter estimation and management performance when spatial heterogeneity is present (Punt 2019). For example, in the MSE of *Thunnus thynnus* , the population is divided into eastern and western stocks, which are characterized by distinct growth models and length–weight relationships, reflecting fundamental differences in life‐history traits between regions (Peterson et al. 2022).

In contrast, current management and assessment frameworks for jumbo squid in the southeastern Pacific have yet to explicitly incorporate spatial structure. Roa‐Ureta and Wiff (2025) submitted a stock assessment for jumbo squid to the South Pacific Regional Fisheries Management Organization (SPRFMO) based on a generalized depletion model, in which biomass estimates are highly sensitive to mean monthly body weight. However, in this assessment, mean body weights for both Asian and Peruvian fleets were derived from industrial fishing data without spatial stratification. As a result, small‐bodied individuals from equatorial waters (often < 1 kg) and large individuals from mid‐ to high‐latitude waters (which may exceed 40 kg) were effectively averaged together, potentially introducing substantial and difficult‐to‐quantify bias into biomass estimates. Moreover, for a semelparous species such as jumbo squid, in which individuals spawn once and die shortly thereafter, spatial variation in age and size at maturity directly influences the timing of post‐spawning mortality, with important consequences for estimates of spawning biomass and stock productivity. This issue is particularly critical for age‐structured assessment models, yet no existing assessment framework adequately captures spatial variation in maturation and post‐spawning mortality for squid populations. Our results highlight the need to develop spatially explicit, age‐structured assessment models for jumbo squid, in which spatial heterogeneity in growth, maturation, and mortality processes is explicitly incorporated to support more robust and biologically realistic management.

## 5 Conclusion and Limitations

Our study reveals that climate exposure at different developmental stages exerts spatially heterogeneous effects on growth in jumbo squid. By integrating age‐structured growth modeling with life stage–specific ENSO exposure, we show that growth responses to climate variability are strongly dependent on both developmental timing and spatial context. Cold ENSO conditions during early ontogeny generally enhanced growth potential, whereas warmer conditions became increasingly favorable at later life stages, reflecting ontogenetic shifts in thermal tolerance, energy allocation, and reproductive investment. These findings highlight the importance of adopting a life‐cycle perspective when interpreting climate–growth relationships in highly migratory marine species. Importantly, our results underscore that treating jumbo squid as a spatially homogeneous population may obscure biologically meaningful variation in growth and maturation, with direct implications for stock assessment and climate‐resilient fisheries management. Our study provides new insight into how climate variability interacts with life‐history processes to shape growth dynamics in short‐lived, highly migratory marine species.

Nevertheless, several data‐related considerations should be acknowledged when interpreting these results. Jumbo squid have been reported to exhibit two to three phenotypic groups, which may coexist and mix within Peruvian–Chilean waters (Yu et al. 2025). The presence of individuals with different growth and maturation strategies may therefore partly explain the greater variability in growth observed in the Southern Group. However, the mechanisms underlying the formation of these phenotypic groups remain unresolved, and immature individuals cannot be reliably assigned to a specific group based on the available biological characteristics. Consequently, the single Schnute curve fitted to the Southern Group should be interpreted as an average regional growth trajectory and may not fully capture growth differences among underlying phenotypic groups. Future studies based on larger, cohort‐resolved datasets and independent criteria for phenotypic‐group assignment are needed to evaluate group‐specific growth trajectories.

An additional limitation is that the study focused on female individuals. Since growth rates, body size, and maturation schedules may differ between sexes in jumbo squid, the spatial growth patterns and life stage–specific ENSO associations identified here should be interpreted as applying specifically to females and should not be generalized directly to males or to the species as a whole. Future studies incorporating both sexes are needed to determine whether males exhibit comparable spatial growth patterns and ontogenetic responses to ENSO variability.

## Author Contributions

**Mingfeng Jiang:** conceptualization (equal), data curation (equal), software (equal), supervision (equal), validation (equal), visualization (equal), writing – original draft (equal), writing – review and editing (equal). **Fan Zhang:** conceptualization (equal), methodology (equal), project administration (equal), resources (equal), writing – review and editing (equal). **Sisong Dong:** visualization (equal), writing – original draft (equal), writing – review and editing (equal). **Jun Yu:** methodology (equal), project administration (equal), validation (equal). **Ming Shao:** resources (equal), software (equal), supervision (equal). **Xinjun Chen:** conceptualization (equal), data curation (equal), formal analysis (equal), funding acquisition (equal), investigation (equal), methodology (equal), supervision (equal).

## Conflicts of Interest

The authors declare no conflicts of interest.

## Supporting information


**Figure S1:** Monthly variation in sample size across regions.
**Figure S2:** Distribution of Niño 1 + 2 SSTA values represented in the Equatorial Group and Southern Group.
**Figure S3:** Time series of Nino 1.2 SSTA (a), Nino 1.2 SST (b), equatorial region SST (c), and southern region SST (d).
**Figure S4:** Distribution of normalized growth residuals of the equatorial group and southern group.
**Table S1:** Overview of jumbo squid samples collected among sampling years.
**Table S2:** Performance of von Bertalanffy growth models with the parameter $t_{0}^{\mathrm{vB}}$ retained.
**Table S3:** Structure and performance of generalized additive models.

## Data Availability

The data that support the findings of this study are openly available in figshare at https://doi.org/10.6084/m9.figshare.32964092.
