# Supplementary material for: Spatially Heterogeneous Responses of Body Growth to El Niño‐Southern Oscillation Signal During Different Life Stages in Jumbo Squid (Dosidicus gigas)
Source: Ecol Evol. 2026 Aug 6;16(8):e74148. doi: 10.1002/ece3.74148 (PMC13445568; doi:10.1002/ece3.74148)
Supplement: Supplementary file 1 [file ECE3-16-e74148-s001.docx]

Tab.S1 Overview of jumbo squid samples collected among sampling years

| Year | Equatorial group | | | Southern group | | |
| --- | --- | --- | --- | --- | --- | --- |
|  | Number | Mantle length  (mm) | Age(month) | Number | Mantle length  (mm) | Age(month) |
| 2009 | 180 | 205-429 | 4.3-9.6 | 441 | 220-594 | 5.7-18.8 |
| 2019 | 112 | 53-165 | 2-5.5 | 65 | 172-581 | 4.3-17 |
| 2020 | 182 | 120-400 | 3.1-8.3 | 129 | 196-580 | 4.8-16.6 |
| 2023 | 46 | 210-505 | 4.8-12.6 | 82 | 425-635 | 10.1-19.3 |
| 2024 | 54 | 209-248 | 3.6-5.8 | 124 | 130-273 | 3.6-7.5 |

Tab. S2 Performance of von Bertalanffy growth models with the parameter $t_{0}^{vB}$ retained

| Model name | Curve type | Random effect | AIC | BIC |
| --- | --- | --- | --- | --- |
| MS01 | von Bertalanffy | $\mathrm{NA}$ | 14138 | 14164 |
| MS02 | von Bertalanffy | $L_{\infty}^{vB}$ | 13925 | 13951 |
| MS03 | von Bertalanffy | $k^{vB}$ | 13923 | 13950 |
| MS04 | von Bertalanffy | $t_{0}^{vB}$ | 13933 | 13959 |
| MS05 | von Bertalanffy | $L_{\infty}^{vB}+k^{vB}$ | 13927 | 13964 |
| MS06 | von Bertalanffy | $L_{\infty}^{vB}+t_{0}^{vB}$ | 13923 | 13960 |
| MS07 | von Bertalanffy | $k^{vB}+t_{0}^{vB}$ | 13924 | 13961 |
| MS08 | von Bertalanffy | $L_{\infty}^{vB}+k^{vB}+t_{0}^{vB}$ | 13943 | 13995 |

Tab.S3 Structure and performance of generalized additive models

| Model with Niño 1.2 SSTA lag (month) | Fixed effect | | | | *Deviance explained* | adj R^2^ |
| --- | --- | --- | --- | --- | --- | --- |
|  | *p_EG_* | N_EG_ | *p_SG_* | N_SG_ |  |  |
| 0 | <0.05 | 574 | <0.01 | 841 | 9.31% | 0.076 |
| 1 | <0.01 | 574 | <0.01 | 841 | 9.29% | 0.076 |
| 2 | <0.01 | 574 | <0.01 | 841 | 9.43% | 0.077 |
| 3 | <0.05 | 570 | <0.01 | 841 | 8.55% | 0.069 |
| 4 | 0.12 | 563 | <0.01 | 833 | 12.1% | 0.10 |
| 5 | <0.1 | 538 | <0.01 | 799 | 16.4% | 0.14 |
| 6 | <0.1 | 351 | <0.01 | 720 | 11.1% | 0.086 |
| 7 | 0.55 | 165 | <0.01 | 687 | 3.99% | 0.01 |
| 8 | 0.58 | 59 | 0.11 | 555 | 9.96% | 0.058 |

Notes: N denotes the number of samples used in the model.

Fig. S1 Monthly variation in sample size across regions

Fig. S2 Distribution of Niño 1+2 SSTA values represented in the Equatorial Group and Southern Group

Fig. S3 Time series of Nino 1.2 SSTA (a), Nino 1.2 SST(b), equatorial region SST (c), and southern region SST (d).

Notes: The shaded areas represent the sampling periods.

Fig. S4 Distribution of normalized growth residuals of the equatorial group and southern group
